# Severe drug-induced repetitive behaviors and striatal overexpression of VAChT in ChAT-ChR2-EYFP BAC transgenic mice

**DOI:** 10.3389/fncir.2014.00057

**Published:** 2014-05-28

**Authors:** Jill R. Crittenden, Carolyn J. Lacey, Tyrone Lee, Hilary A. Bowden, Ann M. Graybiel

**Affiliations:** Department of Brain and Cognitive Sciences and McGovern Institute for Brain Research, Massachusetts Institute of TechnologyCambridge, MA, USA

**Keywords:** amphetamine, dopamine, acetylcholine, striatum, striosome, stereotypy, drug addiction

## Abstract

In drug users, drug-related cues alone can induce dopamine release in the dorsal striatum. Instructive cues activate inputs to the striatum from both dopaminergic and cholinergic neurons, which are thought to work together to support motor learning and motivated behaviors. Imbalances in these neuromodulatory influences can impair normal action selection and might thus contribute to pathologically repetitive and compulsive behaviors such as drug addiction. Dopamine and acetylcholine can have either antagonistic or synergistic effects on behavior, depending on the state of the animal and the receptor signaling systems at play. Semi-synchronized activation of cholinergic interneurons in the dorsal striatum drives dopamine release via presynaptic nicotinic acetylcholine receptors located on dopamine terminals. Nicotinic receptor blockade is known to diminish abnormal repetitive behaviors (stereotypies) induced by psychomotor stimulants. By contrast, blockade of postsynaptic acetylcholine muscarinic receptors in the dorsomedial striatum exacerbates drug-induced stereotypy, exemplifying how different acetylcholine receptors can also have opposing effects. Although acetylcholine release is known to be altered in animal models of drug addiction, predicting whether these changes will augment or diminish drug-induced behaviors thus remains a challenge. Here, we measured amphetamine-induced stereotypy in BAC transgenic mice that have been shown to overexpress the vesicular acetylcholine transporter (VAChT) with consequent increased acetylcholine release. We found that drug-induced stereotypies, consisting of confined sniffing and licking behaviors, were greatly increased in the transgenic mice relative to sibling controls, as was striatal VAChT protein. These findings suggest that VAChT-mediated increases in acetylcholine could be critical in exacerbating drug-induced stereotypic behaviors and promoting exaggerated behavioral fixity.

## Introduction

Acetylcholine is a key intercellular signaling molecule that is released from neurons in the central and peripheral nervous systems as well as from non-neuronal cell types such as immune and epithelial cells (Grando et al., 2007). Imbalances in CNS acetylcholine have been documented in neurologic disorders including Alzheimer’s disease and Parkinson’s disease, and also in drug addiction. Enzymes required for acetylcholine synthesis (ChAT, choline acetyltransferase), break-down (AChE, acetylcholinesterase) and vesicular packaging (VAChT) are particularly abundant in the striatum (Graybiel et al., 1986; Zhou et al., 2001), a subcortical brain region that is important for motor and motivational control and for habit formation (Jog et al., 1999; Graybiel, 2008; Yin et al., 2009). Cholinergic interneurons comprise only 1–2% of the total number of striatal neurons but their processes, along with cholinergic input fibers from brainstem nuclei (Dautan et al., 2014), span the striatum (Graybiel et al., 1986; Kawaguchi, 1992). Furthermore, cholinergic interneurons are thought to correspond to the tonically active neurons (TANs) that undergo semi-synchronous patterns of fire-pause-rebound activity upon presentation of learned or salient sensory cues (Kawaguchi, 1993; Aosaki et al., 1995; Matsumoto et al., 2001; Inokawa et al., 2010; Schulz et al., 2011; Zhao et al., 2011; Doig et al., 2014). The activity of these interneurons is controlled by intrinsic membrane activity as well as a variety of inputs, including excitatory inputs from the cerebral cortex (Reynolds and Wickens, 2004; Doig et al., 2014) and the sensory-responsive parafascicular nucleus of the thalamus (Lapper and Bolam, 1992), local inhibitory input (Gonzales et al., 2013; Doig et al., 2014), and modulatory inputs from cholinergic and dopaminergic fibers (Aosaki et al., 1994; Dautan et al., 2014). All together, these data are consistent with the notion that the cue-related activity of cholinergic interneurons of the striatum serves to re-bias action selection driven by cortico-basal ganglia circuits and their thalamic links (Minamimoto et al., 2009; Ding et al., 2010).

Cholinergic interneurons are frequently located at the borders between striosomes (a.k.a. patches) and matrix, two striatal compartments that have different input-output connections (Gerfen, 1984; Jimenez-Castellanos and Graybiel, 1989; Langer and Graybiel, 1989; Eblen and Graybiel, 1995; Kincaid and Wilson, 1996; Fujiyama et al., 2011; Watabe-Uchida et al., 2012; Gerfen et al., 2013) and that are impacted differentially by psychomotor stimulants (Graybiel et al., 1990; Canales and Graybiel, 2000; Capper-Loup et al., 2002; Horner et al., 2012; Jedynak et al., 2012) and disease (Crittenden and Graybiel, 2011). Preferential disruption of striosomes by toxins or genetic targeting influences the severity of drug-induced stereotypy (Tappe and Kuner, 2006; Liao et al., 2008; Murray et al., 2013). Moreover, ablation of cholinergic interneurons in the striatum blocks the drug-induced striosome-to-matrix gene induction ratio (Saka et al., 2002) and can increase drug-induced stereotypy (Aliane et al., 2011). Together, these data suggest that the cholinergic system mediates interactions between the two striatal compartments (Miura et al., 2008), and the balance between drug-induced hyperlocomotion and restricted, repetitive behaviors (Canales and Graybiel, 2000).

Overexpression of VAChT augments vesicular loading and release of acetylcholine *in vitro* (Song et al., 1997). Moreover, transgenic mouse models that carry multiple copies of *Slc18a3*, the gene encoding VAChT, show an increase in evoked release of acetylcholine in hippocampal slices (Nagy and Aubert, 2012; Kolisnyk et al., 2013b). Transgenic mouse and rat lines that are engineered to drive gene expression in cholinergic cells typically carry exogenous copies of the cholinergic gene locus (Eiden, 1998) with an inactivated *Chat* gene but an intact *Slc18a3* gene. Accordingly, ChAT-ChR2-EYFP BAC mice, which were selected for high-level expression of channelrhodopsin in cholinergic neurons (Zhao et al., 2011), have been shown to overexpress VAChT (Kolisnyk et al., 2013b). Evaluation of ChAT-ChR2-EYFP BAC mice demonstrated that they have normal metabolic rate and baseline locomotor activity but reduced performance in tests for attention, spatial memory, cue-guided memory and working memory (Kolisnyk et al., 2013b). Deletion of VAChT in the prefrontal cortex of mice also disrupts cognitive function, as reflected by reduced reversal learning and attention-task performance (Kolisnyk et al., 2013a). Thus, both hypofunction and hyperfunction of VAChT are associated with impairments in cognitive function.

Here, we show that ChAT-ChR2-EYFP BAC mice have elevated striatal VAchT and abnormally severe confined stereotypies when treated with high doses of D-amphetamine. By contrast, they showed mild hypersensitivity to low doses of D-amphetamine and their behavioral responses to saline injection were relatively normal. These data are consistent with the proposal that the regulation of acetylcholine release is especially important for balancing the response to extreme dopamine stimulation.

## Materials and methods

### Mice

The Committee on Animal Care at the Massachusetts Institute of Technology approved all procedures. ChAT-ChR2-EYFP BAC mice were genotyped from tissue assayed by Transnetyx, Inc. for the presence of *EYFP*. ChAT-ChR2-EYFP BAC transgenic mice (Zhao et al., 2011) were obtained from Prof. Guoping Feng on a C57BL/6J genetic background and crossed to a line on a mixed FVB/N and 129S4 background. Offspring were intercrossed to maintain the line by crossing *EYFP*-positive mice to *EYFP*-negative mice at every generation such that mice homozygous for the transgene were never generated. For the data reported here, ChAT-ChR2-EYFP BAC hemizygous mice were compared to BAC-negative, sibling wildtype mice. Transgenic and control mice were tested in parallel by an experimenter blinded to genotype. All experimental mice were male and group-housed with sibling controls under a standard light-dark cycle (lights on at 7 am and off at 7 pm), with free access to food and water. Mice were between 3–11 months of age at the time of testing.

For the viral vector experiment to evaluate cholinergic neuropil, male mice from the Cre knock-in line, B6;129S6-ChAT<tm2(Cre)Lowl>/J (Rossi et al., 2011), were obtained from Jackson Laboratories and used directly for experimentation.

### Intracerebral viral injection

Adeno-associated virus (rAAV5EF1a-DIO-hChR2(E123T/T159C)-mCherry) was packaged and purified by the Gene Therapy Center Vector Core at The University of North Carolina at Chapel Hill and estimated by dot blot to be at a concentration of 4X10e12 virus molecules/ml.

Mice were anesthetized by injection (i.p., 10 ml/kg) with a mixture of ketamine (120 mg/kg) and xylazine (16 mg/kg) in saline. Mice were mounted onto a stereotactic frame and small burr holes were made bilaterally (AP = 0.9 mm and ML = −1.9 mm and +1.9 mm, relative to bregma). A NanoFil microsyringe (World Precision Instruments) was lowered to deliver 0.5 µl of virus solution at each of two sites on each side of the brain (DV = 2.0 mm and 2.7 mm), at a rate of 0.1 µl/min. Eight weeks after surgery, mice were transcardially perfused and brain sections were obtained for immunohistological examination as described below.

### Immunolabeling and histology

Mice were deeply anesthetized with Euthasol (pentobarbital sodium and phenytoin sodium from Virbac AH Inc.) and perfused transcardially with 15 ml of saline followed by 60 ml of 4% paraformaldehyde in 0.1 M NaKPO_4_, pH 7.4. Brains were post-fixed overnight in 4% paraformaldehyde and then cryoprotected by submersion overnight in 20% glycerin in 0.1 M NaKPO_4_, pH 7.4. Frozen, 24-µm thick, transverse brain sections were cut on a sliding microtome. Immunoreactivity was assessed by standard methods. Briefly, free-floating sections were incubated with primary antisera (anti-VAChT AB1588 from Millipore, 1:100 dilution; anti-ChAT AB144P from Millipore, 1:200 dilution; polyclonal anti-CalDAG-GEFI, 1:5,000 dilution Crittenden et al., 2004). For immunofluorescence, sections were incubated with secondary antibodies coupled to ALEXA594 or ALEXA488 (Invitrogen Corp., anti-rabbit, 1:250 dilution) and then were mounted and coverslipped with Vectashield media (Vector Laboratories). For non-fluorescent immunohistochemistry, sections were incubated with a biotinylated secondary antibody (Vector Laboratories, anti-guinea pig and anti-goat, 1:500 dilution) the signal was amplified and visualized by the Vectastain Peroxidase ABC System (Vector Laboratories), and then sections were mounted and coverslipped with Eukitt (Electron Microscopy Sciences). Brightfield images were obtained on an Olympus BX61 microscope, and confocal images were obtained on a Nikon C2 microscope. EYFP and ALEXA488 fluorophores were excited with a 488 nm solid-state laser and emission was transmitted through a 560 nm short pass filter plus a 510 +/− 42 nm band pass filter. mCherry and ALEXA594 were excited with a 561 nm solid-state laser and emission was transmitted through a 648 nm short pass filter plus a 593 +/− 20 nm band pass filter. High-resolution confocal images were made by summing 5 µm (Figures 2D–F) or 7.5 µm (Figures 2G–I) stacks of images taken at 0.5 µm intervals. Images were processed and analyzed with Fiji software (Schindelin et al., 2012). To ensure that the fluorescence signal from cholinergic neuropil was within a striosome, and not derived from matrix tissue above or below the image plane, care was taken only to stack images in which the CalDAG-GEFI immunoreactivity was consistently low within striosomal borders.

**Figure 1:**
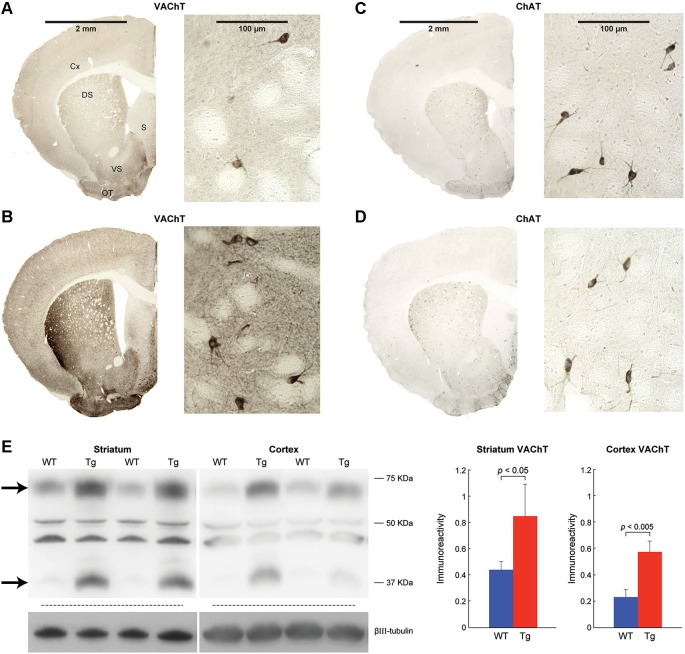
**VAChT is overexpressed in the striatum and cerebral cortex of ChAT-ChR2-EYFP BAC mice**. VAChT **(A,B)** and ChAT **(C,D)** immunoreactivity in coronal brain hemi-sections from a control mouse **(A,C)** and a hemizygous ChAT-ChR2-EYFP BAC mouse **(B,D)**. Corresponding high magnification images at right show cholinergic interneurons located in the dorsomedial striatum. Cx: cerebral cortex, DS: dorsal striatum, VS: ventral striatum, S: septum, OT: olfactory tubercle. **(E)** Immunoblots and corresponding graphs for VAChT expression in wildtype mice (WT) and sibling ChAT-ChR2-EYFP BAC mice (Tg). Arrows point to VAchT-specific bands. Bar graphs represent immunoreactivity based on densitometry of the upper VAChT-specific band, relative to neuron-specific βIII-tubulin in wildtype (blue, *n* = 3) and ChAT-ChR2-EYFP BAC (red, *n* = 3) mice. Values shown are mean + SEM.

**Figure 2:**
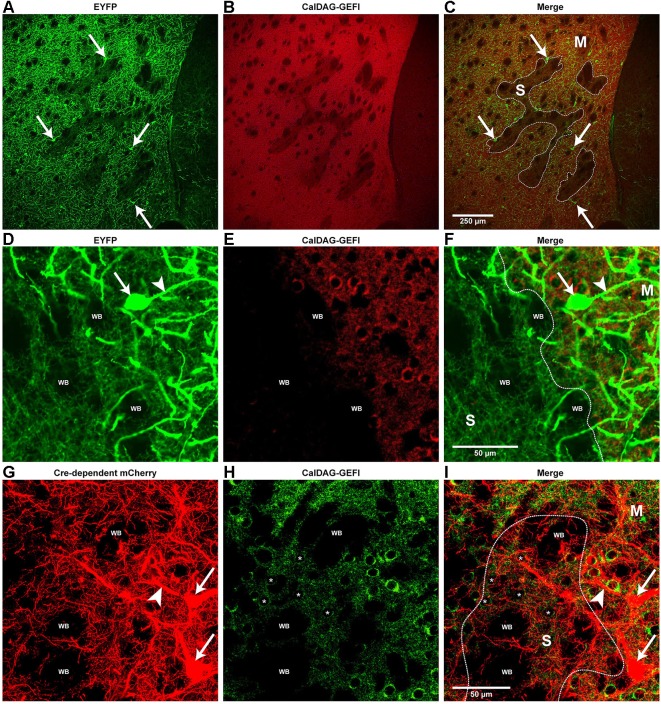
**Cholinergic interneuron processes are differentially enriched in striosome and matrix compartments of the striatum. (A–F)** Confocal images of cholinergic somata (arrows in **A,C,D** and **F**) and processes (green fluorescence) in the dorsomedial striatum of a ChAT-ChR2-EYFP BAC mouse. Striosomes are identified by low immunoreactivity for the matrix marker CalDAG-GEFI (red immunofluorescence in **B,C,E** and **F**). Borders between striosomes and matrix are designated by white lines in **C** and **F**. **(D–F)** High-magnification confocal images of a striosome-matrix border, showing thick, sparsley spiny processes (arrowheads in **D** and **F**), assumed to be dendrites, that are mostly in the matrix compartment (right half of panels). Very thin, EYFP-positive processes with varicosities, assumed to be cholinergic axons, appear equally abundant in both striosomes (left half of panels) and matrix. **(G–I)** High-magnification confocal images of the dorsal striatum from a ChAT-Cre knock-in mouse with Cre-dependent mCherry (red fluorescence) to label cholinergic interneurons (arrows in **G** and **I**). CalDAG-GEFI immunofluorescence (green) defines the matrix compartment. The medium-sized cell bodies with low CalDAG-GEFI immunoreactivity (asterisks in the nuclei of example striosome cells in **H** and **I**) are within a striosome (approximate border outlined in white). Thick cholinergic cell processes (arrowhead in **G** and **I**) are abundant in the matrix and fine processes with varicosities are dense in both the matrix and striosomes. S: striosome; M: matrix; WB: White matter bundles.

### Immunoblotting

Mice were sacrificed by cervical dislocation, and the striatum and overlying cerebral cortex were dissected on a cold plate prior to freezing on dry ice and storage at −80°C. To prepare tissue lysates, frozen tissue was homogenized in ice-cold modified RIPA buffer (50 mM Tris pH 8.0, 150 mM NaCl, 1% Triton X-100, 0.1% sodium dodecyl sulfate, 1% NaDeoxycholate) with protease inhibitor, sodium fluoride, activated sodium orthovanadate and PMSF and centrifuged at 16,000 × g for 10 min to pellet insoluble material. The protein concentrations of supernatants were determined by bicinchoninic acid assays (Pierce). For detection of VAChT, lysates were not boiled prior to resolving proteins by SDS-PAGE. Gel-resolved proteins were transferred to PVDF membrane by using the Invitrogen iBlot. Immunoblotting was accomplished by standard methods. Blots were incubated overnight with antisera against VAChT (139103 from Synaptic Systems, 1:500 dilution). Blots were rinsed and incubated with horseradish peroxidase-coupled secondary antibody (Santa Cruz Biotechnology, Inc. anti-rabbit, 1:5,000 dilution) prior to immunodetection with Immobilon Western (Millipore) according to the manufacturer’s instructions. Blots were subsequently incubated with anti-β-tubulin III (T8578 from Sigma-Aldrich Co., 1:10,000 dilution) and horseradish peroxidase-coupled secondary antibody (Santa Cruz Biotechnology, Inc. anti-mouse, 1:10,000) to normalize total protein loading, as described in the Statistics subsection below.

### Drugs

D-amphetamine (Sigma) was prepared fresh daily by dissolving in saline, and mice were injected with 10 ml/kg (i.p.) for doses of 2.5 mg/kg/day or 7.0 mg/kg/day.

### Behavior evaluation

Locomotor activity was measured using an activity monitoring system and TruScan software (Coulbourn Instruments). Mice were administered saline or drug individually in the activity monitors, which consisted of 25 cm square × 40 cm high arenas surrounded by Plexiglas walls. The floor consisted of a removable plastic drop pan that was cleaned between sessions. A sensor ring, housing 16 infrared beams to detect horizontal movements, surrounded the arena. The animal position was measured every 100 ms, and the software calculated the distance traveled in 5-min bins.

Mice were placed into the activity monitors and given 60 min to habituate prior to injection. Following injection, mice were placed back in the monitors for an additional 85 min of data collection. On days 1–3, mice were injected with saline for habituation to the treatment and environment. On days 4–10, mice were treated with D-amphetamine. Mice were then given 7 days of drug wash-out, with no treatment or handling, prior to a final D-amphetamine challenge treatment. Each mouse received the same dose of D-amphetamine on each day, and separate cohorts of mice were used for the low- and high-dose D-amphetamine treatment experiments. To test for sensitization to the injection procedure itself, one cohort of D-amphetamine (7.0 mg/kg) treated mice was given a challenge dose of saline, 12 days after the D-amphetamine challenge. Mice were videotaped while in the monitors on saline day 1, D-amphetamine treatment day 1, and on the challenge day. Video recordings were 2-min long each, and they were taken at 50 and 80 min after injection. An experimenter blinded to genotype scored the videotapes using a keyboard scoring system with the public domain software JWatcher™, version 1.0 (University of California, LA, CA, USA, and Macquarie University, Sidney, Australia).^1^ Individual keys were assigned to score resting, grooming, locomotion, rearing, sniffing air, sniffing/licking floor, sniffing/licking wall, no sniffing/licking and highly confined stereotypy.

### Statistics

To quantify immunoblot signals, VAChT immunoreactive bands were selected in ImageJ software (Schneider et al., 2012), and their density was measured and normalized to neuron-specific tubulin bands on the same blot. Results were averaged across three independent brain samples and compared by two-tailed Student’s *t*-tests.

**Figure 3:**
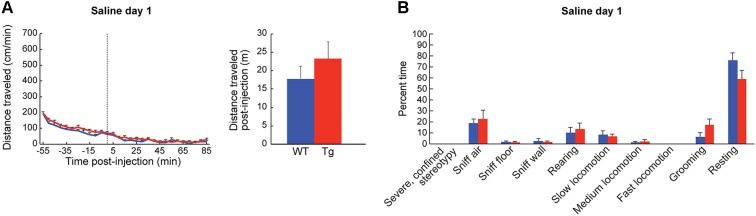
**Baseline locomotor measurements are normal in ChAT-ChR2-EYFP BAC mice. (A)** Distance traveled (mean + SEM) by wildtype (blue, *n* = 13) and ChAT-ChR2-EYFP BAC (red, *n* = 15) mice in an open-field chamber across time, around injection (dotted line) of saline at time 0. The total distance traveled during an 85-min post-injection period is shown in the bar graph. **(B)** Observational ratings of behaviors, averaged for 50 and 80 min post-injection observation points, were equivalent in transgenic mice and sibling controls.

**Figure 4:**
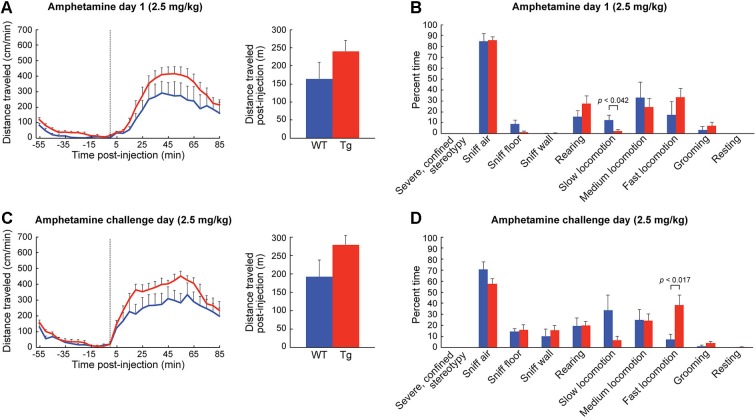
**ChAT-ChR2-EYFP BAC mice show slightly increased motoric responses to low-dose D-amphetamine. (A)** Distance traveled (mean + SEM) in an open-field chamber on the 1st day of D-amphetamine injection (time 0, dotted line) for wildtype (blue, *n* = 6) and ChAT-ChR2-EYFP BAC (red, *n* = 9) mice. The total distance traveled post-injection (85 min) is shown in the bar graph. **(B)** Observational rating of behaviors, averaged for 50 and 80 min post-injection time-points, on D-amphetamine day 1. **(C)** Distance traveled measurements and **(D)** observational ratings on the challenge day of D-amphetamine treatment.

**Figure 5:**
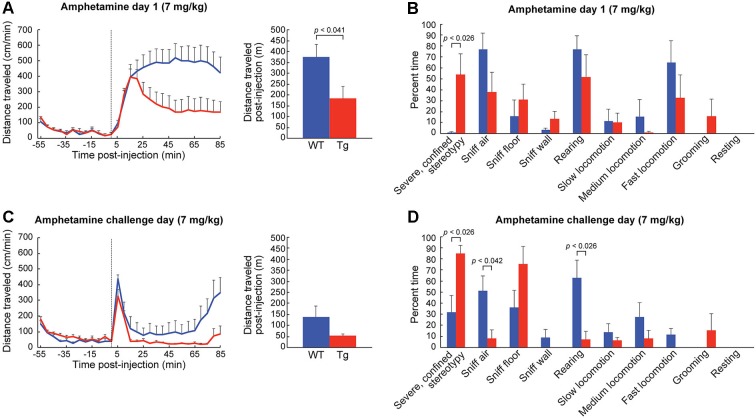
**Confined stereotypies are dramatically elevated in ChAT-ChR2-EYFP BAC mice treated with high-dose D-amphetamine. (A)** Distance traveled (mean + SEM) in transgenic mice (red, *n* = 6) and in wildtype controls (blue, *n* = 6) following the injection (dotted line) of high-dose D-amphetamine on day 1. Total distance traveled after the injection (bar graph) is significantly lower for the transgenic mice. **(B)** Observational rating of behaviors on day 1 showed a significant increase in severely confined stereotypies in transgenic mice relative to sibling controls. **(C)** Distance traveled measurements and **(D)** observational ratings on the challenge day of high-dose D-amphetamine treatment.

Distance traveled and rearing activity graphs are shown as the mean + standard error of the mean for each genotype cohort of mice. Summed distance traveled data (bar graphs in Figures 3–5) from the Truscan recording system were compared between genotypes by unpaired, two-tailed Student’s *t*-tests. Stereotypy scores between genotype cohorts were compared by Mann-Whitney *U*-tests. Significance criteria were set at *p* < 0.05.

## Results

### Overexpression of VAChT in the striatum of ChAT-ChR2-EYFP BAC transgenic mice

VAChT protein products were elevated in striatum of ChAT-ChR2-EYFP BAC transgenic mice, as illustrated by immunohistochemistry in brain slices (Figures 1A,B) and by immunoblot quantitation (Figure 1E). VAChT immunoreactivity was observed in the cell bodies of cholinergic interneurons in both genotypes and in small puncta throughout the striatum, consistent with VAChT function in cell bodies and nerve terminals of cholinergic neurons. Differentially intense VAChT immunoreactive bands were observed at ~70 kDa and ~40 kDa, approximating the size of products that were previously confirmed to be missing in lysates from VAChT knockout mice (Nagy and Aubert, 2012). The intermediate size bands, which were not elevated in the BAC transgenic mice, were presumed to be nonspecific. Immunoreactivity for ChAT appeared to be similar in striatum of ChAT-ChR2-EYFP BAC transgenic mice, relative to controls (Figures 1C,D). Our results are consistent with reports that, relative to controls, mRNA for VAChT is elevated 20-fold and mRNA for ChAT is unchanged in the striatum of ChAT-ChR2-EYFP BAC transgenic mice (Kolisnyk et al., 2013b).

### Cholinergic neuropil distribution in striosome and matrix compartments

The overexpression of enhanced yellow fluorescent protein (EYFP) in cholinergic neurons of the ChAT-ChR2-EYFP BAC transgenic mice provided an opportunity to detect fine neuronal processes that could not be fully labeled with traditional methods (Matsuda et al., 2009). To observe how cholinergic neuropil was distributed relative to the striosome and matrix compartments, we labeled striatal sections from the ChAT-ChR2-EYFP BAC transgenic mice with red immunofluorescence for the matrix marker, CalDAG-GEFI (Kawasaki et al., 1998) to compare to the pattern of EYFP fluorescence. We observed that EYFP-positive cholinergic interneuron somata (arrows in Figures 2A,C,D and 2F) were frequently in close proximity to one another and to striosomal borders (Figures 2A–F) and, although predominantly in the matrix, were occasionally located within a striosome. Thick, sparsely-spiny, EYFP-positive processes emanated from EYFP-positive cell bodies and were presumed to be dendrites (arrowhead in Figures 2A,F). These putative cholinergic interneuron dendrites sometimes appeared to cross striosome-matrix borders, but were nevertheless more prevalent in the matrix than in the striosomes (Figures 2A–F). In low-magnification images of the medial striatum, striosomes appeared as EYFP-poor zones (Figure 2A), presumably owing to the matrix enrichment of these EYFP-positive dendrites, as well as matrix-preferring EYFP-positive cholinergic fibers from brainstem nuclei (Dautan et al., 2014). By high-resolution confocal microscopy, however, very thin, EYFP-positive processes with varicosities, presumed to be axons, could be visualized in both matrix and striosome compartments (Figures 2D–F), as indicated in previous ChAT immunostaining methods analyzed with light microscopic methods (Graybiel et al., 1986), but even more clearly seen here with confocal microscopy. Thus the cholinergic neuropil of the striatum, although intense in the matrix compartment, is differentiated, with potential acetylcholine releases sites abundant in both striosomes and matrix and dense dendritic arbors in the matrix compartment.

To determine whether the distribution pattern of cholinergic neuropil that we observed might be unique to the ChAT-ChR2-EYFP BAC transgenic mice, we examined cholinergic interneurons in a ChAT-Cre knock-in line (Rossi et al., 2011) that does not have duplication of the cholinergic gene locus. To label the cholinergic interneurons in this line, we injected, into the striatum of the mice, an adeno-associated virus that carries a Cre-dependent gene encoding the red fluorophore, mCherry (described in Section Materials and Methods). Eight weeks after viral injection, we obtained brain sections and double-labeled them by green immunofluorescence for the matrix marker, CalDAG-GEFI. Compared to the ChAT-ChR2-EYFP BAC mice, we observed fewer cholinergic interneurons labeled by this method, and the cholinergic neuropil appeared less dense in most regions. However, the compartmental distribution of labeled processes was similar in the two lines (Figures 2G–I). In the ChAT-Cre knock-in line, the thick, mCherry-positive processes (presumed dendrites) crossed compartment borders but were more abundant in the CalDAG-GEFI-positive zones (matrix), than in the striosomes (CalDAG-GEFI-poor). By contrast, the very fine mCherry-positive processes with varicosities (presumed axons) appeared similarly dense between the striosome and matrix compartments (Figures 2G–I). These results again suggest that inputs to the cholinergic interneuron dendrites are enriched in the striatal matrix (Herkenham and Pert, 1981; Graybiel et al., 1986; Sadikot et al., 1992; Fujiyama et al., 2006; Raju et al., 2006), whereas potential axon-terminal release sites from cholinergic interneurons are dense in both striosomes and matrix.

### Severe drug-induced stereotypy in ChAT-ChR2-EYFP BAC transgenic mice

To test whether the ChAT-ChR2-EYFP BAC transgenic mice had abnormal responses to psychomotor stimulants, we measured locomotion and stereotypy in transgenic mice and sibling controls treated with low or high doses of D-amphetamine. To measure locomotion each mouse was placed into an activity monitor fitted with infrared photobeams to monitor mouse movements and calculate distance traveled. To measure drug-induced stereotypy, we video-recorded the mice for 2 min, at 50 and at 80 min post-injection and a rater blinded to genotype scored the frequency and duration of each behavior observed.

To habituate the mice to the activity chamber and also to gather baseline behavior data, we injected the mice with saline (10 ml/kg, i.p.) for three consecutive days prior to drug treatment. We found no differences in behavior between the ChAT-ChR2-EYFP BAC transgenic mice and sibling controls injected with saline in the novel chamber (Figures 3A,B). In response to the first injection of low-dose D-amphetamine (2.5 mg/kg, i.p.), the ChAT-ChR2-EYFP BAC transgenic mice showed a tendency for a greater locomotor response than their sibling controls, but this effect did not reach statistical significance (Figure 4A). The time spent in slow versus fast locomotion was significantly less for the transgenic mice, reflecting the tendency for their increase in distance traveled (Figure 4B). To test for drug sensitization, we treated the mice for an additional 6 days with the same dose of D-amphetamine followed by a 7-day drug washout period with no treatment, and then measured their response to a drug challenge at the same dose-level. On the challenge day, both transgenic and control mice showed evidence of sensitization in that they began locomotion much sooner after drug injection than they did on the first day (Figure 4C). The total distance traveled did not appear increased on challenge day relative to the first day of treatment, which is likely related to the increase in pausing for wall-sniffing in the sensitized mice (Figure 4D compared to 4B). On the challenge day, the transgenic mice showed evidence of drug hyperresponsivity based on significantly more time spent in fast locomotion than controls (Figure 4D). Altogether, the ChAT-ChR2-EYFP BAC mice showed slight hypersensitivy to low-dose D-amphetamine, both in their response to acute treatment and after repeated treatment inducing drug sensitization.

The response of the transgenic mice to high doses of D-amphetamine (7.0 mg/kg) was strikingly different from that of their wildtype siblings. The distance traveled scores of the transgenic mice started to fall sharply at the 20 min post-injection time point (Figure 5A), as they began to engage in severe and confined stereotypic behaviors such as sniffing the floor or the wall in the corners of the monitors (Figure 5B). After repeated high-dose D-amphetamine treatment, both transgenic and control mice developed sensitized responses to the drug, indicated by their short latencies to the onset of locomotion after drug-injection (Figure 5C) and increase in severe stereotypy (Figure 5D), relative to day 1. In summary, the ChAT-ChR2-EYFP BAC mice had more severe confined stereotypic behavior, in both the naïve and the drug-sensitized state, than the corresponding control mice (Figures 5B,D).

**Figure 6:**
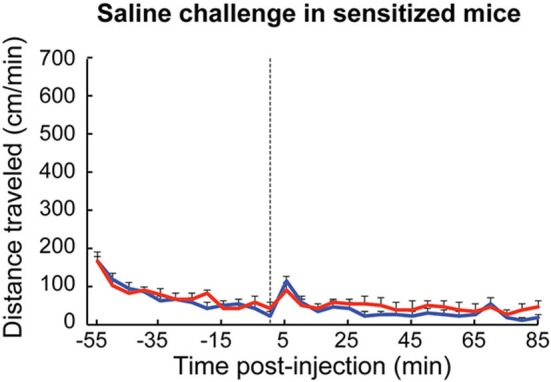
**Responses of amphetamine-sensitized mice to saline injection were equivalent between genotypes**. Wildtype (blue, *n* = 6) and transgenic (red, *n* = 6) mice that were previously treated with repeated D-amphetamine (7 mg/kg) injections showed similar sensitized locomotor responses to saline injection (dotted line).

Considering that cholinergic interneurons are reported to be responsive to learned and salient cues, we also tested whether the mice that had been sensitized to high-dose D-amphetamine would show a sensitized locomotor response to saline injection only. After drug sensitization, both ChAT-ChR2-EYFP BAC and control mice showed a sharper response to saline injection than they did prior to drug sensitization (Figure 6, compared to Figure 3A), but there were no apparent differences between genotypes. Thus, the transgenic mice did not exhibit blockade either of behavioral sensitization to D-amphetamine injection itself or of the capacity to become sensitized to cues associated with injection of the drug.

## Discussion

Our findings point to an abnormal behavioral phenotype in BAC transgenic ChAT-ChR2-EYFP mice in which the mice exhibit excessively severe amphetamine-induced stereotypy. It is likely that this phenotype derives from overexpression of VAChT in these mice. Consistent with the finding that *Slc18a3* transcription is elevated (Kolisnyk et al., 2013b), we observed significantly higher VAChT protein immunolabeling in the striatum and cerebral cortex of hemizygous transgenic mice than in their littermate controls, as determined both by immunoblotting and by immunohistochemistry. Thus, this BAC mouse line is likely to have increased stimulated acetylcholine release in the striatum, based on findings of increased release of acetylcholine in hippocampal slices from this line and a second, similar line (B6.eGFPChAT) (Nagy and Aubert, 2012; Kolisnyk et al., 2013b). VAChT immunoreactivity appeared to be particularly abundant in the lateral striatum, which contains cholinergic interneurons as well as cholinergic afferent fibers from the pedunculopontine nucleus (Dautan et al., 2014), and excitatory inputs from sensorimotor regions of the thalamus (Lanciego et al., 2004) and neocortex (Kincaid and Wilson, 1996). Notably, this VAChT-enriched region appears to be near to the ventrolateral zone that induced the highest levels of oral stereotypy in a mapping study made by local amphetamine injections across the striatum (Dickson et al., 1994).

The VAChT overexpression finding is congruent with chromosomal insertions of multiple copies of the ChAT-ChR2-EYFP BAC construct (Zhao et al., 2011), in which the first part of the *Chat* coding region was replaced by the ChR2-EYFP cassette, but the* Slc18a3* gene, which is located within intron 1 of *Chat* (Eiden, 1998), was not altered. A similar BAC construct design has been used to generate numerous transgenic rodent lines in order to drive gene expression in cholinergic cells. Such transgenic lines include a channelrhodopsin line (Ren et al., 2011; Zhao et al., 2011), a ribosomal L10a marker TRAP line (Doyle et al., 2008; Heiman et al., 2008), fluorescent reporter lines (Gong et al., 2003), a tau-GFP line (Grybko et al., 2011), mouse Cre lines (Gong et al., 2007), and a rat Cre line (Witten et al., 2011). ChAT BAC lines selected for high transgene expression levels would be expected to have correspondingly high expression of VAChT. Knock-in lines in which the transgene is targeted to the endogenous ChAT locus (Rossi et al., 2011), or BAC transgenic lines in which VAChT is specifically inactivated (Ting and Feng, 2014), could be the exceptions. It is well-recognized that the genetic background (Thomsen and Caine, 2011), as well as the sex, age and housing conditions of mice influence their responses to psychomotor stimulants. The phenotype of the BAC mice described here highlights the importance of controlling for the possibility that BAC transgenic mice carry extra copies of genes or have a gene mutation caused by chromosomal insertion of the BAC. Abnormal responses to cocaine were discovered for a Drd2-EGFP BAC mouse line as well (Kramer et al., 2011), although this phenotype is reported to be sensitive to genetic background and is dependent on homozygosity for the BAC insertion (Chan et al., 2012).

The fluorophore overexpression in the ChAT BAC transgenic mice, and virus-injected ChAT-Cre knock-in mice, permitted us to observe the cholinergic neuropil in fine detail. The dendrites that originated from the fluorophore-labeled cholinergic interneurons were sparsely spiny and were more prevalent in the matrix compartment than in the striosomes of the dorsomedial striatum (Graybiel et al., 1986). This finding complements evidence that the thalamic parafascicular nucleus preferentially targets the striatal matrix, and is a major source of input to the dendritic shafts of cholinergic interneurons (Herkenham and Pert, 1981; Lapper and Bolam, 1992; Sadikot et al., 1992; Fujiyama et al., 2006; Raju et al., 2006). The compartmentalized distribution of EYFP-positive dendrites was most obvious in the striosome-rich medial striatum, a region specifically implicated in the cholinergic regulation of drug-induced stereotypy (Aliane et al., 2009). In contrast to the matrix-enrichment of dendrites from the cholinergic interneurons, very thin, EYFP-positive cholinergic processes, with abundant varicosities typical of axons, extended throughout both the matrix and striosomes, a finding strongly extending the original observation of this fine intra-striosomal neuropil (Graybiel et al., 1986). This apparent innervation of striosomes by the fine fibers of striatal cholinergic neurons contrasts with the reported minimal innervation of striosomes by inputs arising from cholinergic neurons in the brainstem, which strongly and preferentially innervate the matrix compartment (Dautan et al., 2014). If verified by further co-labeling of these two sources of cholinergic input to the striatum, these results together would suggest that direct cholinergic innervation of striosomes likely arises specifically from the cholinergic interneurons.

AChE, the main degradative enzyme of acetylcholine, is enriched in the matrix in humans (Graybiel and Ragsdale, 1978), bringing up the further possibility that acetylcholine signaling is more transient in the matrix than in striosomes. Although such differential AChE distributions are scarcely visible in the rodent striatum, preferential striosomal expression of c-Fos is induced by high-dose amphetamine treatment in both rodents and monkeys *in vivo* (Graybiel et al., 1990; Canales and Graybiel, 2000; Saka et al., 2004; Horner and Keefe, 2006; Jedynak et al., 2012) and this compartmentalized pattern is disrupted by ablation of cholinergic interneurons (Saka et al., 2002). Together with our immunfluorescence findings, these observations suggest that acetylcholine release could directly and differentially influence the striosome and matrix compartments.

The ChAT-ChR2-EYFP BAC transgenic mice suffered severe stereotypy, both after acute administration of D-amphetamine and after repeated administration of this drug. There is abundant evidence that changes in acetylcholine levels in the striatum are linked to drug-induced stereotypy. *In vivo* microdialysis studies in behaving rats show that acute, binge methamphetamine treatment protocols that induce high levels of stereotypy lead to changes in acetylcholine release in the dorsal striatum, relative to pre-drug levels. Rats that are given prolonged, repeated drug treatments that result in tolerance to the stereotypy-inducing effects of methamphetamine have less repression of acetylcholine release (Kuczenski and Segal, 2001). By contrast, the levels of acetylcholine in the ventral striatum are not different in rats that show tolerance versus sensitization to drug-induced stereotypy (Kuczenski and Segal, 2001), supporting the idea that drug-induced stereotypy is related to activity in the dorsal striatum. Other microdialysis studies, however, suggest that rats exhibiting a sensitized stereotypic responses to amphetamine have an *increase* in striatal acetylcholine (Bickerdike and Abercrombie, 1997), rather than a decrease. The reasons for these opposing effects of psychomotor stimulants on acetylcholine levels are unclear (Kuczenski and Segal, 2001), but the studies nevertheless converge to show a strong correlation of behavioral stereotypy with changes in striatal acetylcholine.

This relationship between acetylcholine and drug-induced stereotypy is still not understood at a mechanistic level. Pharmacologic studies indicate that stereotypy can be influenced by postsynaptic muscarinic receptors as well as presynaptic nicotinic acetylcholine receptors. One potential mechanistic link comes from the fact that activation of presynaptic β2 nicotinic acetylcholine receptors, under certain conditions, can enhance dopamine release from terminals in the dorsal striatum (Zhou et al., 2001; Perez et al., 2009; Threlfell et al., 2012) and that drug-induced stereotypy is associated with co-activation of D1- and D2-type dopamine receptors (Capper-Loup et al., 2002). Moreover, repeated nicotine administration induces stereotypy in rats and also enhances behavioral responses to cocaine (Collins and Izenwasser, 2004). Notably, two studies show that DHβE administration in mice reduces sensitization of stereotypies in response to repeated drug administration, but does not change the stereotypy in response to first-time drug exposure (Karler et al., 1996; Metaxas et al., 2012), suggesting that β2 nicotinic receptors are important for sensitization of stereotypy, but not for the acute stereotypic response. Collectively, these findings raise the possibility that ChAT-ChR2-EYFP BAC transgenic mice are “born sensitized” by virtue of having molecular abnormalities that are similar to those in sensitized animals, and that this predisposition biases them toward exhibiting an increased response to their first D-amphetamine exposure, without being so severe as to occlude further sensitization.

VAChT is reported to be up-regulated in post-mortem striatal samples from human methamphetamine users (Siegal et al., 2004), suggesting that humans exposed to drugs of abuse might have abnormal acetylcholine release akin to that in rodent models of drug addiction (Bickerdike and Abercrombie, 1997; Kuczenski and Segal, 2001) and in transgenic mice with VAChT overexpression (Nagy and Aubert, 2012; Kolisnyk et al., 2013b). Considering the findings reported here of increased responses to a habit-forming psychomotor stimulant in mice that ovexpress VAChT, dysregulation of VAChT in humans might be directly related to drug addiction. Reversible AChE inhibitors are prescribed for medical conditions of reduced acetylcholine function including myasthenia gravis and Alzheimer’s disease (Nair and Hunter, 2004). AChE inhibitors are also under investigation for the treatment of methamphetamine addiction (De La Garza et al., 2012) and motor tics in Tourette syndrome (Cubo et al., 2008). Despite the widespread use of compounds that reduce the breakdown of acetylcholine, there is incomplete information about the effects of augmenting acetylcholine release. The distinction between these two approaches for elevating acetylcholine may an important one, considering that the phasic release of acetylcholine is thought to be important for cognitive attention (Sarter et al., 2009) and that there are profound differences between tonic and phasic release of dopamine (Goto et al., 2007; Schultz, 2007). Whether stimulation or overexpression of VAChT could be beneficial for particular medical conditions remains to be tested.

The functions of acetylcholine in the striatum depend upon a multitude of factors including the differential activation and dynamics of acetylcholine receptor subtypes, the striatal region under study, and the firing patterns of the dopamine-containing neurons innervating the striatum (Morris et al., 2004; Perez et al., 2009; Threlfell and Cragg, 2011; Zhang and Sulzer, 2012). Moreover, these neuromodulatory influences are themselves associated with functions in numerous neural circuits and cell types. We highlight the severe stereotypic behavior of the ChAT-ChR2-EYFP BAC transgenic mice because of its striking potential reflection of the power of the striatal cholinergic system to influence repetitive behaviors induced by habit-forming drugs. This strong overexpression phenotype suggests that the cholinergic system is poised to regulate responses to intense dopaminergic stimulation, conditions engendered by drug use. Cell signaling mechanisms across the body are relevant to an animal’s response to drugs of abuse, and how the loss of frontal control contributes to addiction (Feil et al., 2010) may be related to how cue-sensitivity, motivation, and memory are integrated (Flagel et al., 2009).

## Conflict of interest statement

The authors declare that the research was conducted in the absence of any commercial or financial relationships that could be construed as a potential conflict of interest.

## Footnotes

^1^http://www.jwatcher.ucla.edu/
